# Nanometer- and angstrom-scale characteristics that modulate complement responses to nanoparticles

**DOI:** 10.1016/j.jconrel.2022.09.039

**Published:** 2022-09-27

**Authors:** S. Moein Moghimi, Hajira B. Haroon, Anan Yaghmur, Dmitri Simberg, Panagiotis N. Trohopoulos

**Affiliations:** a School of Pharmacy, Newcastle University, Newcastle upon Tyne NE1 7RU, UK; b Translational and Clinical Research Institute, Faculty of Health and Medical Sciences, Newcastle University, Newcastle upon Tyne NE2 4HH, UK; c Colorado Center for Nanomedicine and Nanosafety, University of Colorado Anschutz Medical Center, Aurora, CO, USA; d Department of Pharmacy, Faculty of Health and Medical Sciences, University of Copenhagen, 2100 Copenhagen Ø, Denmark; e Translational Bio-Nanosciences Laboratory, Department of Pharmaceutical Sciences, Skaggs School of Pharmacy, University of Colorado Anschutz Medical Center, Aurora, CO, USA; f CosmoPHOS Ltd, 77 Tsimiski Street, GR-54622 Thessaloniki, Greece

**Keywords:** Antibodies, C1q, Complement system, Dendrimers, Factor H, Mannose-binding lectin, Nanoparticles

## Abstract

The contribution of the complement system to non-specific host defence and maintenance of homeostasis is well appreciated. Many particulate systems trigger complement activation but the underlying mechanisms are still poorly understood. Activation of the complement cascade could lead to particle opsonisation by the cleavage products of the third complement protein and might promote inflammatory reactions. Antibody binding in a controlled manner and/or sensing of particles by the complement pattern-recognition molecules such as C1q and mannose-binding lectin can trigger complement activation. Particle curvature and spacing arrangement/periodicity of surface functional groups/ligands are two important parameters that modulate complement responses through multivalent engagement with and conformational regulation of surface-bound antibodies and complement pattern-recognition molecules. Thus, a better fundamental understanding of nanometer- and angstrom-scale parameters that modulate particle interaction with antibodies and complement proteins could portend new possibilities for engineering of particulate drug carriers and biomedical platforms with tuneable complement responses and is discussed here.

## 1. Introduction

**Fig. 1.**
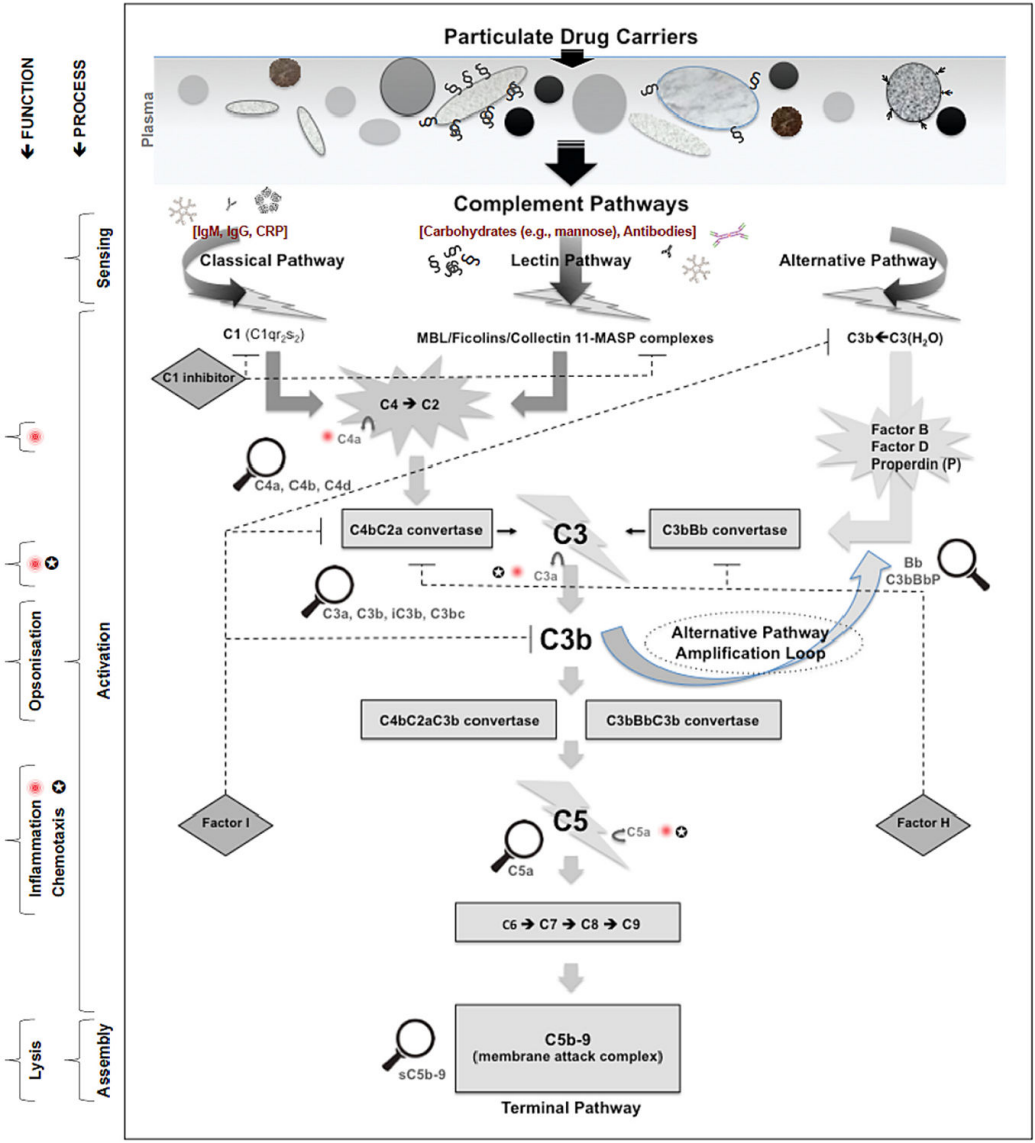
Complement activation pathways. The scheme shows sequential enzymatic steps in classical, lectin and alternative complement activation pathways, key complement regulators (e.g., C1-inhibitor, Factor H, Factor I) and selected complement activation products (C4a, C4b, C4d, C3a, C3b, iC3b, C3bc, Bb, C5a, sC5b-9) that can be monitored by different methodologies. Antibodies such as IgM, IgG and IgA, depending on their isotype and conformation, can trigger complement activation through the three pathways. C-reactive protein (CRP) on binding to nanoparticles might also trigger complement activation through the C1 complex arm of the classical pathway. The binding of complement pattern recognition molecules C1q and collectins (MBL, ficolins and collectin-11) to surfaces triggers activation of their associated proenzymes (C1r and C1s in the case of C1q; MASPs in the case of collectins), resulting in complement activation through classical and lectin pathways, respectively. Alternative pathway turnover occurs through autoactivation of soluble C3 that undergoes slow spontaneous hydrolysis [C3(H_2_O)], or when nascent C3b undergoes nucleophilic attack; for instance, by hydroxyl or amino groups on nanopartiCle surfaces. The first critical stage in complement cascade is the formation of C3 Convertases (C4bC2a and C3bBb) that cleave C3. This results in the liberation of anaphylatoxin C3a and the opsonic fragments C3b and iC3b. The next step is the assembly of C5 Convertases (C4bC2aC3b and C3bBbC3b), which cleaves C5, resulting in liberation of another anaphylatoxin (C5a) and activation of the terminal pathway of complement. On full complement activation the membrane attack complex C5b-9 is formed. In soluble form, C5b-9 is bound to vitronectin (sC5b-9). The figure and the legend are reproduced with permission from [21] with a slight modification.

**Fig. 2.**
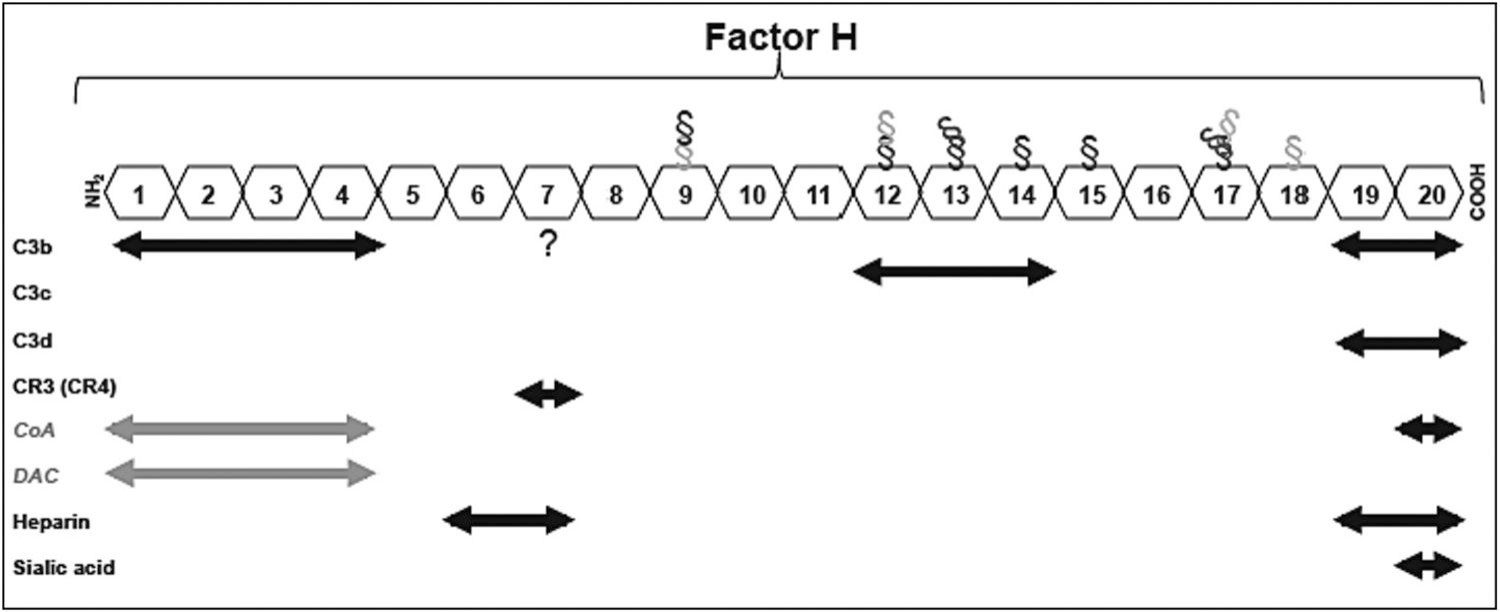
Schematic representation of factor H showing the locations of the complement and polyanion binding sites. Factor H is composed of 20 domains. Factor H also regulates complement activation by possessing both cofactor activity (CoA) for the factor I-mediated C3b cleavage and decay accelerating activity (DAC) against the alternative pathway C3 convertase through domains 1–4. § = glycosylated region; CR = complement receptor.

Our immune system is equipped with diverse arsenal of molecular and cellular elements that recognise and eliminate invaders and damaged self-materials. Among these, the complement system of the innate immunity is a central player [1,2]. Here, opsonisation by the third complement protein (C3) on activation of any of the three established complement pathways (classical, lectin and alternative) aids recognition of many pathogens and particulate drug delivery systems by the mononuclear phagocytic cells for clearance and destruction (Fig. 1) [2]. Many virulent pathogens, however, have developed an array of complex surface strategies to evade complement opsonisation and combat phagocytic recognition. These include the expression of surface projected proteins (e.g., the M protein of group A streptococci) and polysaccharides (e.g., sialohomopolymers such as polysialic acids) that hijack complement regulators such as factor H (Fig. 2), which suppresses C3 opsonisation [3,4]. Some virulent pathogens also attract antibodies in conformations and orientations that cannot trigger complement activation or engage with macrophage Fc receptors [5,6]. Other versatile pathogen-adopted complement evasion strategies include surface expression of proteins that bind to C3, its cleavage products (C3b and iC3b) and the fifth complement protein (C5) [7,8]. These recruitment strategies change C3 and C5 conformations in such a way that prevents their participation in the downstream events of complement cascade (e. g., as in C3 and C5 convertase assembly, respectively). Conceptually, defensive protein- and polysaccharide-based surface strategies also create a protective hydrophilic shell, thereby hampering a direct contact between the pathogen and the phagocyte through repulsive forces [9].

In the field of drug delivery systems, early attempts introduced polymeric nanoparticles that were coated with A-B-A linear and star-shaped block copolymers (otherwise known as Pluronic and Tetronic series) with the vision of mimicking the phagocytic escape properties of virulent pathogens [9,10]. These copolymers adsorb on to the hydrophobic surfaces through their central hydrophobic polyoxypropylene chain (B block). This leaves the hydrophilic polyoxyethylene segments (A blocks) to project from the surface creating a protective dynamic shell surrounding the nanoparticle core. Depending on the conformational cloud of surface projected polyoxyethylene chains, it became clear that nanoparticles can not only resist recognition and ingestion by macrophages, but also the polymer conformation can exert a modulatory effect on the pathway and the extent of complement activation [11,12]. Here, a surface projected “brush-like” polymeric conformation proved more effective in suppressing C3 opsonisation and preventing nanoparticle ingestion by macrophages as compared with corresponding nanoparticles displaying polymers in a “mushroom” conformation [12]. Similar conclusions followed with nanoparticles bearing poly(ethylene glycol) (PEG) and with those decorated with polysaccharide shells [13-15]. Notwithstanding, today human complement opsonisation still prevails with the majority of investigated polymer-coated/grafted nanoparticles, albeit to a lesser extent compared with the pristine surface [1,2,12-15]. However, the steric barrier of the projected polymer chains seemingly interfere with the engagement of the surface deposited opsonic C3 fragments (C3b and iC3b) with complement receptors of the phagocytic cells [2].

Despite these abovementioned developments, complement activation is still problematic from a therapeutic perspective. For instance, complement activation when uncontrolled could induce inflammation and promote disease progression through inflammatory and chemotactic processes [2]. Thus, therapeutic interventions with nanomedicines should consider tailor-made strategies that either minimise or overcome complement activation. From a broader perspective, approaches that eliminate complement activation by nanoparticles may find applications for improved engineering of medical devices such as stents, contact lenses and haemodialysis membrane filters. While considering these, it is imperative to note that mapping nanoparticle- and medical device-mediated complement responses in many preclinical species may not necessarily translate into similar outcomes in humans. Furthermore, major differences exist among different strains of the same species in complement activation processes and these should be taken into account in preclinical studies that involve the prediction of complement responses in humans [1,2,16-21]. Nevertheless, due to such disparities it is necessary to test complement responses in human plasma/sera at all stages of preclinical development and match this with appropriate animal/species models [16,17,21].

Notwithstanding, improved understanding of the dynamic and multifaceted mechanisms that modulate complement responses is a priority for robust development and engineering of immunologically safe nanomedicines for human use. Here we discuss these paying particular attention to the role of nanoparticle curvature and surface pattern periodicity in modulating complement responses.

## 2. The promiscuous role of particle curvature in antibody-directed complement activation

Antibodies play a major role in complement activation, since they can trigger complement cascade through all the three complement pathways, but the modes and the extents of complement activation are dependent on the antibody type, glycosylation and conformation [22-26]. Particle characteristics, notably their size and surface properties, regulate antibody deposition and conformation either directly or through non-specific protein binding to particle surfaces [2,26-30]. For example, a single pentameric or hexameric IgM can trigger complement activation through the classical pathway; however, the efficacy of complement activation is dependent on the conformation of the bound antibody [27]. Indeed, it was the earlier work of Feinstein and Munn that showed IgM adopts a staple-like (strained) conformation on binding to a bacterial flagellum [31]. In the unbound state IgM assumes a planar conformation in solution, but it is the strained form of the IgM that promotes interactions with the classical pathway component C1 complex [32]. To adopt the optimal staple-conformation for accommodating the C1-complex, it has been suggested that IgM must bind to a surface through its Fab arms in an angle of approximately 68° relative to the plane of the Fc regions [27]. Multivalent Fab interactions with surface epitopes on 100–250 nm particles (corresponding to a curvature range of 0.02–0.008 nm^−1^) seem to sufficiently strain IgM and initiate complement activation [27]. Theoretically, IgM straining is expected to be even more prominent as particle sizes decrease and approaches the cross-sectional diameter of IgM (which is approximately 40 nm) [27]. However, it appears that such steeply curved surfaces are not very efficient in triggering the classical pathway of the complement system through IgM binding [27]. This is presumably due to topological constrains, which dramatically limit multivalent engagement of all Fab regions with the surface motifs, leading to insufficient IgM straining. However, if IgM has very high affinity for the surface motifs, then IgM straining could become significant and complement activation prevails. This might explain inter-individual variability seen in complement activation with 50 nm nanoparticles [27]. It further appears that with larger particles (≥500 nm in diameter), but still dependent on epitope density and orientation, again, IgM does not necessarily adopt the optimal staple-like conformation for efficient C1 complex binding [27]. Thus, relatively larger particles are less efficient in activating the classical pathway of the complement system as compared with particles of 100–250 nm in diameter.

Monomeric IgG has a very low affinity for C1q, but an antigen-driven IgG clustering could lead to the formation of IgG hexamers that bind to C1q with high avidity [33,34]. This results in activation of the classical pathway. However, the composition of N-glycans in the C_H_2 domain of IgG further plays a critical role in modulating antibody conformation and its ability to acquire C1q [24]. For example, Fc-domain deglycosylation abrogates complement activation by inhibiting IgG hexamerization via modulation of IgG Fc:Fc interactions, which, in turn, minimises C1q binding [24]. On the other hand, Fc-galactosylation modulates IgG Fc:Fc interactions for antigen-bound IgG and improves C1q binding [24]. In addition to these, antigen binding to IgG hexamers further potentiates complement initiation, suggesting that Fabs impact downstream Fc-mediated events [24]. Studies with particles artificially coated with bovine serum albumin (BSA) as an antigen and subsequently treated with polyclonal anti-BSA IgG highlighted the importance of the particle size as well as the antibody Fc density on the extent of complement activation [28]. Again, complement response was decreased with increasing the particle size (0.5–4.0 μm) for a given Fc density. However, a relatively high density of Fc was necessary to initiate complement activation [28] presumably by triggering the formation of necessary IgG clusters (e.g., hexamers) capable of accommodating C1q. The extent by which these findings apply to smaller particles (≤200 nm in diameter), which are more commonly used in drug delivery applications [1,2,35], remains unclear, since complex nanoscale parameters (e.g., antigen presentation patterns/periodicity) and antibody quality (e. g., binding affinity and sensitivity) regulate surface assembly of IgG molecules into hexamers via Fc:Fc interactions. Thus, a better understanding of inter-related nanoscale parameters is needed to adequately delineate topographies needed for engineering nanoparticles capable of overcoming antibody-dependent complement activation.

**Fig. 3.**
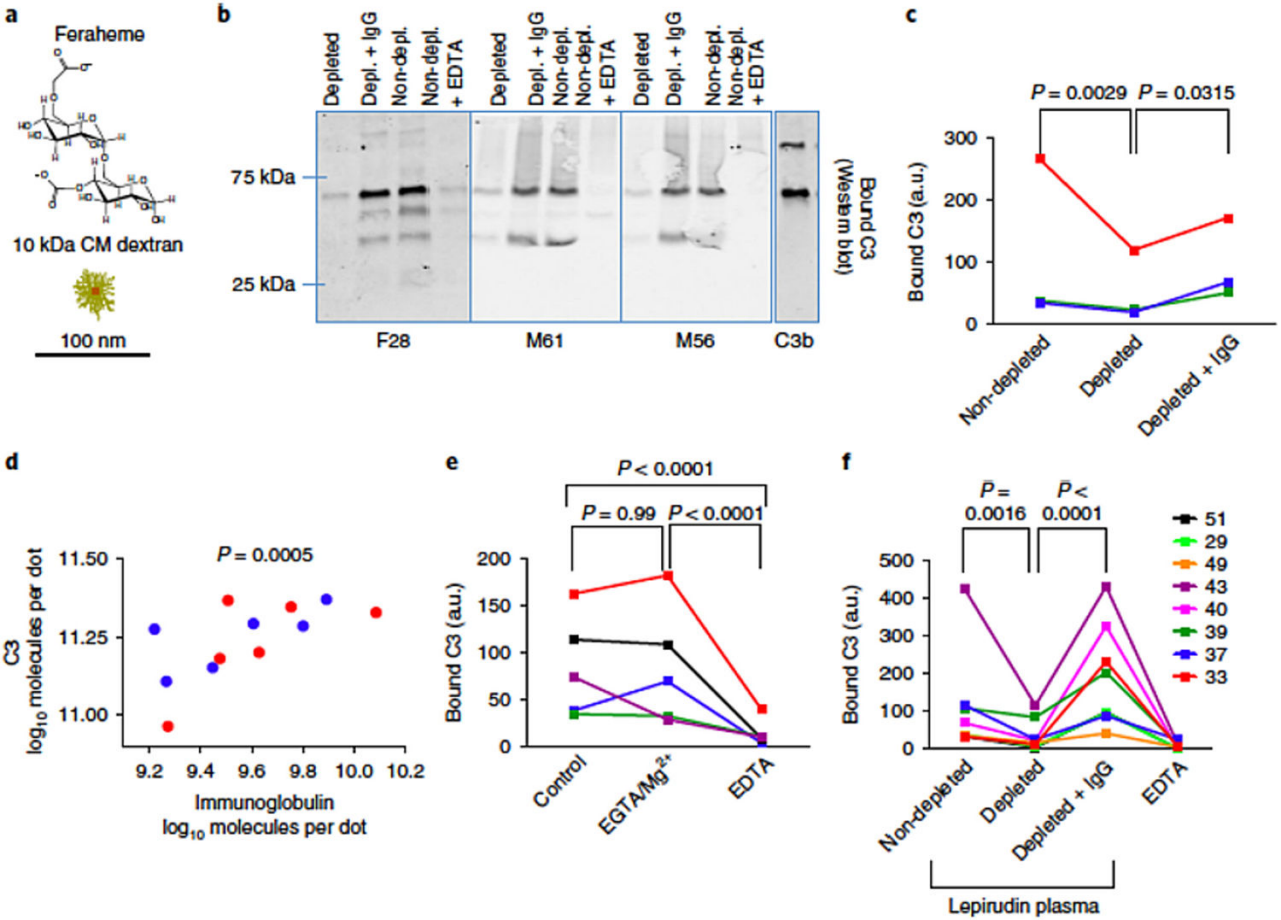
Role of immunoglobulins in efficiency of C3 deposition on Feraheme^®^. (a) Schematic representation of Feraheme structure. CM = Carboxymethyl. (b) Effect of immunoglobulin depletion and reconstitution in plasma of three donors (F28, M61, M56). (c) Effect of immunoglobulin depletion in sera of healthy donors measured with dot blot assay. Each dot represents the mean of three technical replicates per sample. (d) Correlation between levels of immunoglobulins and C3 bound to Feraheme in healthy sera of 12 subjects. (e) Ferahame was incubated in sera with EGTA/Mg^2+^ or EDTA. In (c) and (e) each colour refers to the same individual. Each dot represents the mean of three replicates per sample. (f) Immunoglobulin depletion and reconstitution in lepirudin-anticoagulated plasma from eight breast cancer patients. Each dot represents the mean of three technical replicates per sample. The figure and the legend are reproduced from [30] with permission.

Considering the above discussion, it should also be stressed that the extent of nanoparticle opsonisation by C3 fragments (C3b and iC3b) and macrophage uptake may not necessarily correlate with the observed curvature-dependent differences in the extent of antibody-mediated C1q-dependent classical pathway activation. Generally, macrophages preferably ingest larger C3b opsonised particles than their smaller counterparts, when normalised for the surface area. This is presumably due to more build up of C3b on the surfaces of larger particles (but dependent on the availability of reactive groups that bind covalently to C3b) through the amplification loop of the alternative pathway. Here, curvature might also regulate the affinity of both factor H and factor B, which compete for binding to surface-bound C3b molecules. For instance, a comparable or lower affinity of factor H than factor B for surface-bound C3b on larger particles could shift the balance from regulation to rapid build up of the opsonic C3b. This suggestion might partially explain why relatively larger nanoparticles are more susceptible, at least, to complement receptor-mediated macrophage clearance than their smaller counterparts [2,35], despite their poorer antibody-directed complement activation [27]. Other important factors are the geometrical constraints and the lack of available local surface area on steeply curved surfaces (e.g., ≤50 nm particles), which might limit surface assembly of C3 convertases, since convertases are typically >20 nm in size [29,36]. Furthermore, by considering that a surface-bound C3b could occupy an area of approximately 40 nm^2^ [27,37] only a limited number of C3b molecules can covalently deposit on surfaces of very small particles. This is, in turn, also dependent on the availability of reactive groups for a nucleophilic attack. Even with a successful assembly of C3 convertases on ≤50 nm particles, the bulk of generated C3b molecules are expected to appear in the fluid phase. These C3b molecules will be available for opsonising the nearby particles or macromolecules. This process seems to occur with Feraheme^®^ (colloidal superparamagnetic iron oxide nanocrystals coated with carboxymethyl dextran with an overall diameter of 17–31 nm) [30]. Here, complement activation was strictly antibody (IgG)-dependent but through the alternative pathway (Fig. 3) [30]. Indeed, some forms of human IgG and its F (ab’)2 can directly activate the alternative pathway [26,38,39]. With Feraheme the results showed a stoichiometry ratio of 4 IgG molecules per 1000 Feraheme nanocrystals, which corresponded to the deposition of 150 C3 molecules per 1000 nanocrystals [30]. Therefore, within a population of nanoparticles, only a few nanoparticles promote the assembly of C3 convertases, where a single convertase is capable of cleaving up to 1000 C3 molecules. These convertases, therefore, generate high C3b turnover, which might opsonise some nearby particles presumably through their adsorbed non-specific plasma proteins [30]. The efficiency of this process might be related to the reduced rate of inactivation of the covalently bound C3b to immunoglobulin by factors H and I [22].

In addition, the literature also suggests the existence of antibodies (notably of IgM and IgG class) that bind PEG [40]. Here, the binding of anti-PEG antibodies to PEGylated liposomes can apparently trigger complement activation and promote vesicle clearance from the blood circulation [41]. However, limited structural information is available on the mode of anti-PEG antibody binding to highly flexible PEG conformation. Crystal structure of an anti-PEG antibody Fab clone has revealed an open ring-like sub-structure in the Fab paratope that binds PEG (PEG antigen epitope of 16 monomer subunits) and predominantly is stabilised via Van der Waals interactions along the interior and exterior of the ring paratope surface [42]. However, it is not clear whether an anti-PEG IgM can make multivalent engagement through its Fab regions on PEGylated nanoparticles and attain a functional staple-like conformation to accommodate C1 complex. Likewise, the PEGylated nanoparticle surface must allow formation of anti-PEG IgG clusters (hexamers) of favourable Fc:Fc spacing and interactions for complement activation to proceed. Furthermore, non-specific plasma protein intercalation into the PEG cloud may negatively impact anti-PEG IgG clustering. Considering these, it is likely that the predominant complement response, irrespective of the antibody type (IgM or IgG), is initiated through sufficiently high-affinity interactions resulting in antibody-mediated activation of the alternative pathway, as described previously [30]. In line with this suggestion, one study has, indeed, confirmed a major role for the alternative pathway in anti-PEG antibody-mediated liposomal doxorubicin cargo release [43]. Liposome lysis is an intriguing observation and cryogenic electron microscopy has further confirmed pore formation in the liposome bilayer [43]. These pores are, presumably, generated through the assembly and insertion of the complement membrane attack complex (MAC) into the liposomal bilayer. What remains to be elucidated is how MAC can penetrate through the PEG cloud and reach the liposome bilayer. We speculate a role for the vesicular heterogeneity, where MAC-mediated lysis could be limited to vesicles with low PEG density [44,45], where MAC, due to its large mass, induces PEG cloud rearrangement or collapse. These perturbations might also promote phospholipid-PEG conjugate shedding from the liposome bilayer, a process that substantially reduces the shielding effect of the PEG cloud. The aforementioned liposome lysis studies [43] were done with purposely-generated anti-PEG monoclonal antibodies with high affinity for PEG. However, high titres of naturally occurring high affinity anti-PEG antibodies are rare and assays used for detection and determination of anti-PEG antibodies [40] have rarely examined antibody affinity and antibody-directed complement responses on PEG-decorated nanoparticles of different curvatures.

Studies of Chen et al. [43] also identified a minor role for C1q-dependent complement activation in liposome lysis. It is plausible that this process is antibody-independent, where C1q directly initiates complement activation through electrostatic interactions (e.g., between the cationic C1q A chain and the anionic phosphate oxygen of the phospholipid-mPEG conjugate) and hydrogen bonding (e.g., through the ether oxygen groups of the PEG chains) [46]. Finally, it should be stressed that vesicular aggregation by IgM molecules could also result in complement activation through different pathways (e.g., IgM-mediated lectin pathway activation and alternative pathway activation), which was not investigated in the aforementioned studies, and may further account for rapid clearance of liposome immunoaggregates by mononuclear phagocytes through multifaceted processes.

## 3. From curvature to pattern periodicity

**Fig. 4.**
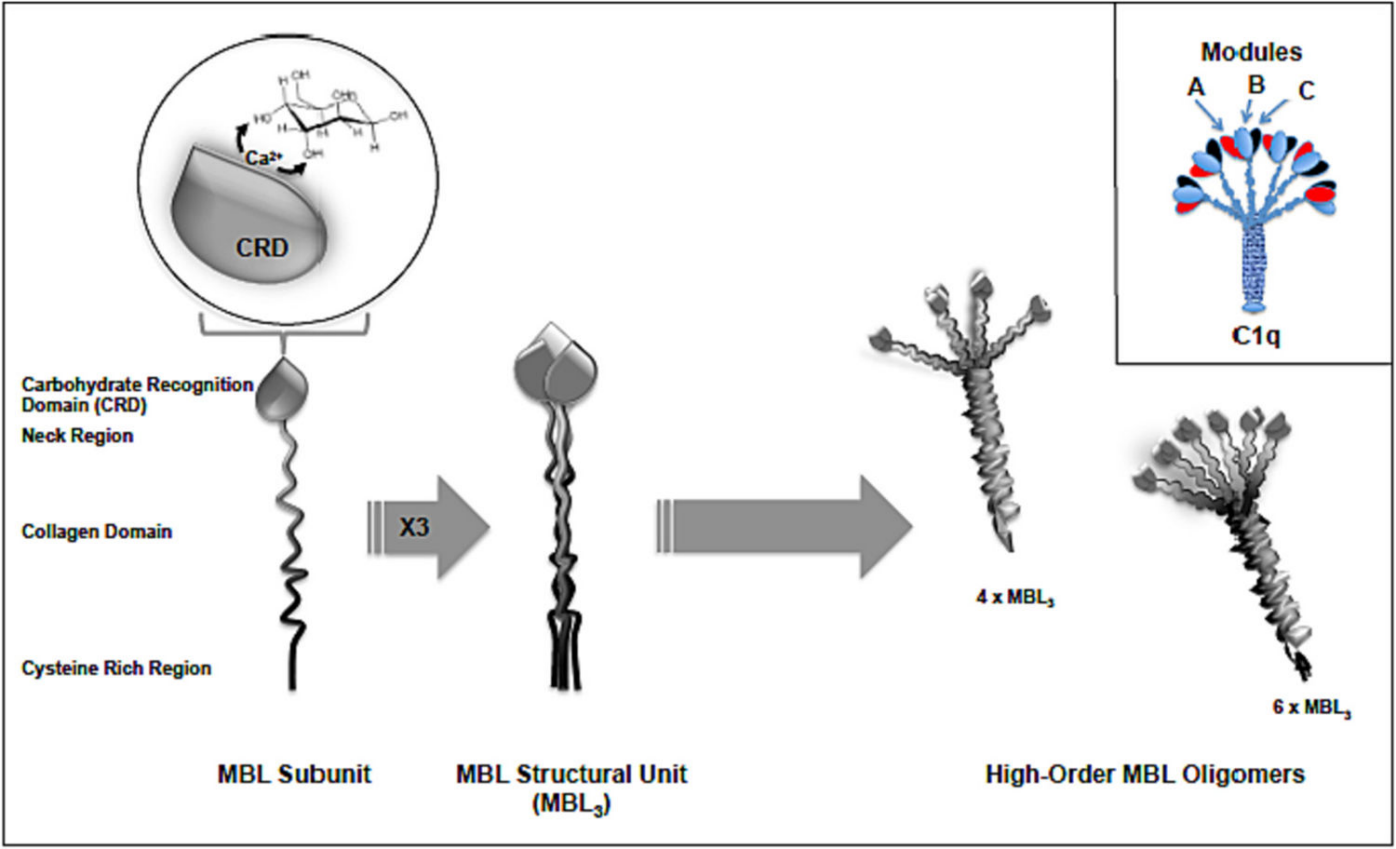
Schematic representation of C1q and mannose-binding lectin (MBL). C1q drawing is reproduced with permission from [2].

Complement pattern recognition molecules (including C1q, MBL and ficolins) have a distinctive sertiform shape (a hub from which the subunits spread out) with many binding sites that recognises different spectrum of ligands [47-52]. For example, C1q is a hexameric protein assembled from six heterotrimeric collagen-like fibres (Fig. 4), each bearing a C-terminal globular head composed of three modules (A, B and C) that recognise clusters of anionic charge or hydrophobic motifs on particle surfaces [47]. Thus, modules A and C show a combination of basic and acidic amino acids over their respective surface, whereas module B shows a predominant expression of cationic amino acids that are thought to participate in the interaction with IgG [47]. There are several hydrophobic residues on the external face of each module, but module C also expresses solvent accessible aromatic amino acids on its equatorial region [47]. While each head of C1q is set at 3–5 nm apart from each other, it is the flexibility of the collagenous stalks that allows the globular heads to spread and appear either in close (as little as 2–3 nm apart) or distant (as much as 15 nm apart) arrangement [53]. This permits recognition of target motifs spaced at intervals of 2–15 nm. The MBL polypeptide consists of a cysteine-rich N-terminal, a collagenous-like region and a calcium-dependent C-terminus domain, which recognises hydroxyl motifs of sugars (e.g., axial hydroxyl groups in D-mannose) (Fig. 4) [48-51]. In the folded state, the collagenous region forms a structural unit of homo trimmers of MBL (MBL_3_) polypeptide chain. These units, in turn, form larger oligomers of 3 to 8 units (3–8 X MBL_3_) and associate themselves with the lectin pathway proteases (MBL-associated serine proteases, MASPs) (Fig. 4) [49-51]. Thus, these structural arrangements and diversity make MBL a highly polydisperse molecule. Each head of MBL (a trimer of neck/carbohydrate recognition domains) has three equivalent target binding sites of 5 nm apart [49]. Again, due to the flexibility of the stalk structures, but depending on the oligomer size, surface binding of MBL involves recognition of patterns with dimensions in the range of 2–20 nm [53,54]. Accordingly, larger oligomers (e.g., 6 X MBL_3_) bind with a high affinity to the surfaces with ligands spaced as wide as 14 nm apart, whereas smaller oligomers (e.g., 3 X MBL_3_) bind with a high affinity to the surfaces with a denser display of ligands (spaced ~5–6 nm apart) [54]. Therefore, the nanometer scale structural features of complement pattern-recognition molecules play important roles in target recognition and modulating complement responses.

**Fig. 5.**
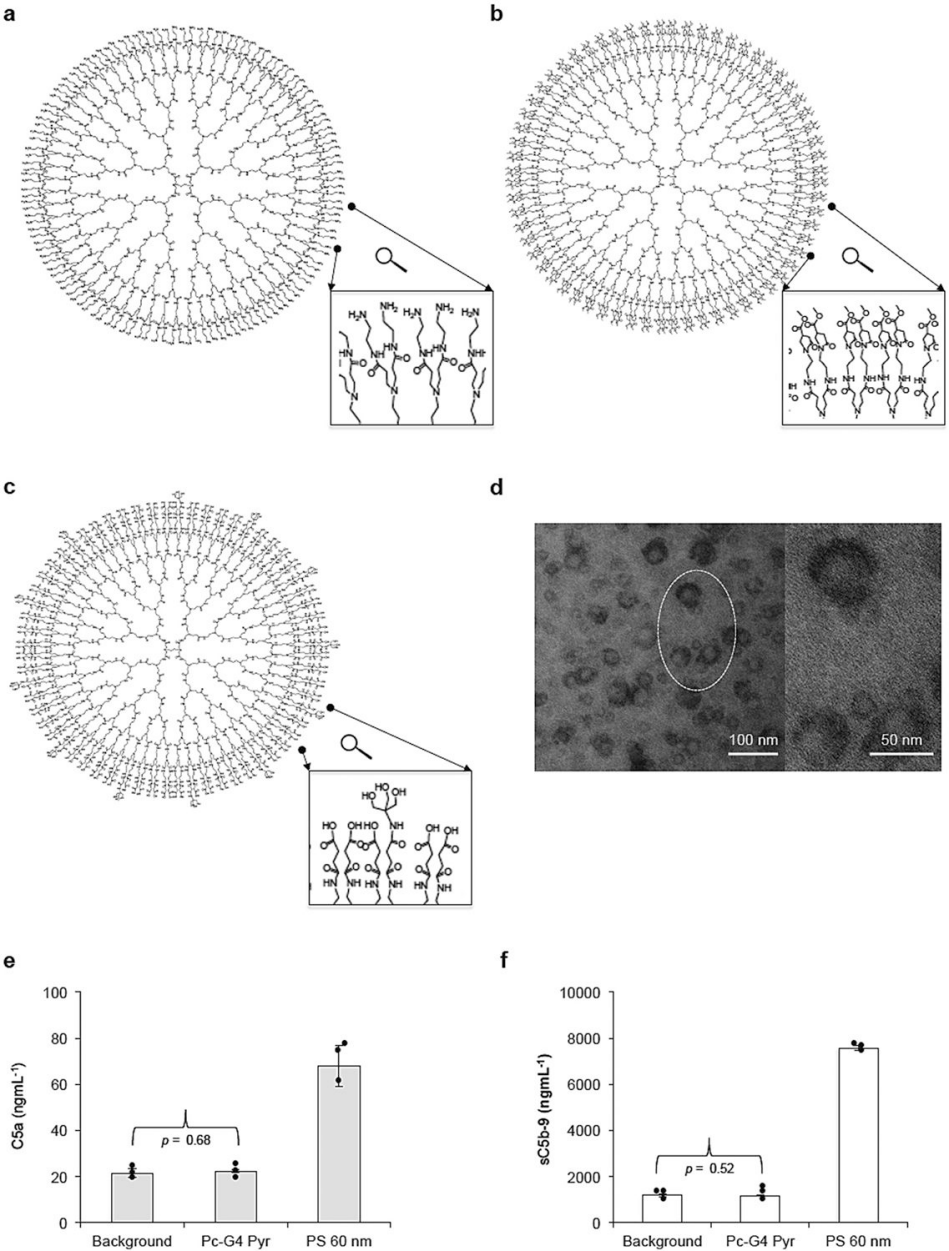
Schematic representation of PAMAM dendrimers and complement activation by dendrimeric platforms. (a–c) Generation 5 PAMAM dendrimers with amine, pyrrolidone and carboxylic acid-Tris end-terminal functionalities. (d) Transmission electron micrograph of generation 4 pyrrolidone-terminated PAMAM dendrimer complexes with phthalocyanine (Pc). Pc is covalently attached to dendrimers. (e & f) Complement responses to pyrrolidone-terminated dendrimer-Pc complexes (Pc-G4 Pyr) (3.5 mgmL^−1^) in a lepirudin-anticoagulated human plasma through measurements of fluid-phase C5a (e) and sC5b-9 (f). A commercially available sulfated polystyrene nanoparticle suspension (PS) of 60 nm was used as positive control for complement activation (PS concentration = 3.5 mgmL^−1^). The figure and the legend are reproduced with permission from [55] with a slight modification.

Because functional binding of complement pattern-recognition molecules to target epitopes is dependent on the ligand periodicity and their nanometer-scale spacing arrangement, entities that display surface patterns below the nanometer-scale spacing arrangement are therefore expected to evade complement. Recently, we tested this hypothesis with early generation poly(amido amine) dendrimers (generation 2–5) [55]. Dendrimers are supramolecular structures with uniform branches displaying precise numbers of surface end-terminal motifs of different functionalities such as amine, carboxyl and pyrrolidone (Fig. 5) [56]. Since dendrimer sizes are controlled in a geometrical fashion, this results in an exponential increase in the number of surface end-terminal motifs. Thus, generation (G) 2–5 dendrimers display 16, 32, 64 and 128 end-terminal motifs, respectively. Considering that G4 and G5 poly (amido amine) dendrimers resemble “hard-spheres” of 4.5 and 5.4 nm [57,58], respectively, a theoretical surface area of ~0.57 nm^2^ is available per end-terminal motif [55]. However, by considering lack of sufficient space near the dendrimer surface, it is likely that some of the end-terminal motifs are back-folded and projected inwards. Thus, dendrimers provide a unique opportunity for testing the role of “Angstrom-scale spacing arrangement (ASSA)” of functional motifs in complement response. Our studies showed that G2–5 poly(amido amine) dendrimers with amine, carboxyl and pyrrolidone end-terminal motif escape sensing by C1q and MBL and do not trigger complement activation through complement pattern-recognition-mediated classical and lectin pathways, respectively [55]. While carboxyl- and pyrrolidone-terminated dendrimers fully evaded complement activation, amine-terminated dendrimers indirectly triggered complement activation [55]. The latter class of dendrimers hitchhiked on a subset of natural IgM glycoforms that bind MBL [55]. However, it is not clear whether the interactions between amine-terminated dendrimer and IgM generate a ligand complex for MBL binding, or induce conformational changes in IgM, leading to exposure of complex oligomannose glycans on the antigen-binding face of the immunoglobulin that bind MBL-MASPs. The former possibility is analogues to the proposed IgM binding to intestinal and/or endothelial neoepitopes, following ischemia/reperfusion [59]. This triggers lectin pathway and contributes to ischemia/reperfusion-induced tissue inflammation and injury through C3 deposition [59]. Thus, amine-terminated poly(amido amine) dendrimer interaction with IgM in vivo may have ramifications in dendrimer pharmacokinetics, biodistribution and safety. For instance, a significant fraction of intravenously injected generation 3 amine-terminated poly(amido amine) dendrimers accumulates in the kidney [60]. Therefore, local dysregulated complement activation by deposited amine-terminated dendrimer-IgM complexes in the kidney may eventuate renal injury.

On the other hand, complement evasion by carboxyl- and pyrrolidone-terminated poly(amido amine) dendrimers is exciting and could open a number of opportunities for engineering of complement evading composite nanomedicines and devices. The motivation came from the observation that larger complexes (in the order of 50 nm) formed between carboxyl- or pyrrolidone-terminated poly(amido amine) dendrimers and large aromatic macrocyclic compounds still evades complement activation (Fig. 5) [55]. Thus, it appears that through ASSA display of surface ligands/functional groups, larger particles (which potentially could engage at least two heads of C1q or MBL) can escape sensing by the complement pattern-recognition molecules. Therefore, it would be intriguing if surface functionalization of particulate drug carriers, implants and medical devices with a monolayer (or stacks) of carboxyl- or pyrrolidone-terminated dendrimers could generate complement-evading surfaces. This further opens plug-and-play strategies in studying the role of pattern periodicity in complement regulation and non-specific protein binding as well as for design of dendrimer-driven multifunctional complement-safe nanomedicines (e. g., for applications in theranostics) and biomedical platforms/devices. We also envisage future attempts in surface engineering of biomedical devices through synthesis of super-hydrophobic dendrimers that repel proteins, which could overcome the possibility of complement activation through non-specific plasma protein deposition.

## 4. Brothers in arms

The proposed ASSA phenomenon also brings curiosity to the microbial systems to study new possibilities by which virulent pathogens evade complement activation. Hijacking of complement regulatory protein factor H (and factor H-like protein 1) by some virulent pathogens through specific receptors and/or molecular mimicry has been an intriguing mechanism in overcoming complement activation and amplification, since factor H is the primary negative regulator of the alternative pathway [3,4,61]. Factor H competes with factor B for binding to C3b but it has a 10-fold increase in affinity towards C3b in the presence of host sialic acid [62,63]. In addition to this, factor H is accelerating the decay of surface-bound complement convertases and act as a cofactor for factor I-mediated C3b fragment inactivation [64]. These regulatory functions of factor H are carried out through 20 homologous complement control protein modules (CCP) (Fig. 2) [61]. For instance, CCPs 1–4 bind C3b and CCPs 19–20 recognise C3b, iC3b and C3d, but CCP 20 also bind sialic acid [61]. Five human factor H-related proteins have also emerged expressing domains homologous to CCPs 6–9 and 18–20, but they lack significant complement inhibitory activity [65]. For instance, factor H-related protein 1 can inhibit C5, whereas protein 2 inhibits the alternative pathway C3 convertase [65]. Factor H–related protein 3 and 4 have been shown to enhance the cofactor activity of factor H [65]. Nonetheless, pathogens that display sialylated glycans and polysialic acids have the advantage of attracting factor H, where the CCP 20 domain of factor H binds to the glycerol side chain and the carboxyl moiety of sialic acid [66]. However, the efficacy of factor H binding, and hence complement escape, is still dependent on the number of available and accessible binding sites on the cell wall. Considering this limitation, it might be possible that cell wall ridges and depressions further display clusters of carboxyl and hydroxyl motifs in ASSA [55] to further escape complement attack independent of factor H-directed molecular crypsis.

**Fig. 6.**
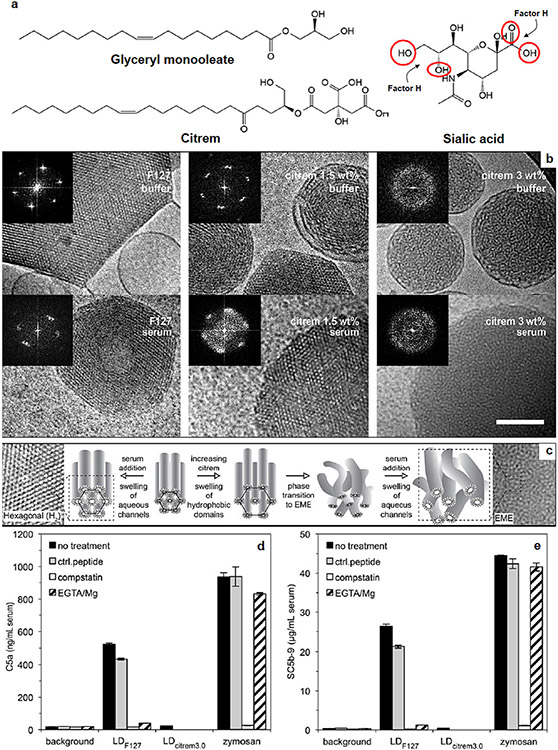
Complement responses towards lyotropic non-lamellar liquid crystalline (LLC) nanodispersions. (a) Structures of glyceryl monooleate, citrem and sialic acid. Circles in sialic acid structure denote factor H binding regions. (b) Cryogenic transmission electron micrographs of LLC nanodispersions from a binary lipid mixture of glyceryl monooleate and medium-chain triglycerides, which have been stabilised either with Pluronic F127 (3 wt%) or citrem (1.5 or 3 wt%). Images show nanodispersions suspended in both buffer and human serum. Inset = the fast Fourier transform analysis of particle interiors. Scale bar = 100 nm. (c) Schematic illustration for the effects of citrem and serum on the nanostructural features of the LLC nanodispersions with representative cryogenic electron micrograph enlargements. (d & e) Complement activation by Pluronic F127-stabilised (LD_F127_) and 3 wt% citrem-stabilised (LD_citrem3.0_) LLC nanodisprsions measured as an elevation of the two end-point complement markers C5a (d) and sC5b-9 (e) in human serum. Zymosan (0.2 mgmL^−1^) was used as positive control for complement activation. Complement activation is exclusively through calcium-sensitive pathways. The figure and the legend are reproduced with permission from [67].

Earlier, we demonstrated the formation of lyotropic non-lamellar liquid crystalline (LLC) aqueous nanodispersions from a binary mixture of glyceryl monooleate and medium-chain triglycerides in the presence of the anionic citric acid ester of monoglycerides (citrem) as a stabiliser (Fig. 6) [67]. Intriguingly, citrem stabilisation prevented activation of the terminal complement pathway. We speculated that the citric acid moiety of citrem, which is in close proximity to the glycerol component of glyceryl monooleate, might generate spatial displays that closely resemble factor H binding sites on sialic acid [67]. In a follow-up study, lamellar (liposomes) and LLC nanodispersions (including cubosomes and hexosomes) formed on mixing citrem and soybean phospholipid activated complement through both calcium-sensitive and alternative pathways in a citrem concentration-dependent manner with more profound complement activation (as a measure of activation of the forth complement protein, C4) with higher citrem levels [68]. However, complement activation did not culminate in activation of the terminal pathway [68]. Since the latter is C5 convertase-dependent, these observations suggest that LLC nanoparticles most likely recruit factor H through carboxylic acid moiety display of specific surface density and periodicity. Likewise, the surface pattern display of citrem-derived carboxylic acid functionality might also explain the extent of calcium-sensitive classical pathway activation through direct C1q recruitment. Factor H recruitment might also account for the observed poor-complement activation properties of other particulate entities with surface display of carboxylic acid and sulfonate functionality [69]. Considering morphological and surface inhomogeneity among lamellar and non-lamellar liquid crystalline nanoparticles in a typical nanodispersion, some nanoparticles may express domains with functional groups in Angstrom-scale proximity and display, and this might further contribute to the complement evading property of the nanoparticle surface.

Our attempt with surface enrichment of PEGylated nanoparticles bearing carboxylic acid functionality at the distal end of the phospholipid-PEG_2000_ conjugate proved unsuccessful in fending off the complement [13]. We attributed this to the partial collapse of the long and flexible PEG_2000_ chains, leading to the formation of a conformational cloud with scattered exposure of carboxylic acid end-groups [13]. On the other hand, PEG-pairing, through incorporation of more rigid short-chain methoxy-PEG_550_, dramatically reduced complement activation [13]. PEG pairing apparently stretches PEG_2000_ chains (i.e., adopting an extended “brush-conformation”) [13]. This PEG stretching might have either generated surface carboxylic acid-rich domains with conformations that recruit factor H and/or displayed carboxylic acid moieties with sufficient periodicity and Angstrom-scale proximity that are not sensed by complement pattern-recognition molecules. In addition to these, coarse-grained molecular dynamic simulations suggested that PEG-pairing, through the aforementioned conformational attributes, minimises statistical protein binding/intercalation [13]. This could be an additional factor limiting (or preventing) sequential processes needed for surface assembly of complement convertases [30]. Considering these notions, our studies with a broad library of LLC nanoparticles have also stressed that the type of linkage between the lipid and methoxy PEG as well as the inner architectural arrangements might further contribute to PEG chain mobility and stretching and minimise complement activation [70]. Therefore, it is plausible that controlled surface PEG stretching could generate nanoscale arrangements that minimise or deter protein binding/intercalation. In line with this suggestion, theoretically, for optimal protein exclusion stretched PEG chains need to be ~1 nm apart [71,72].

## 5. Future prospects

Particle curvature and surface pattern arrangement are two major parameters that modulate complement responses. Emerging findings discussed above strongly indicate that these parameters affect the mode of antibody binding and modulate recruitment of complement pattern-recognition molecules and complement regulators. A better understanding of these mechanisms is needed to accelerate engineering and development of complement-evading nanomedicines and biomedical platforms/devices through integrated combinatorial synthesis and machine learning initiatives. It is also emerging that a small population of engineered nanoparticles (within a typical batch) could potentially be the driving force for the assembly of complement convertases [30]. Therefore, a grand challenge is how to identify and eliminate such a “complement convertase-seed” population. While through careful refinement of particle size and surface characteristics complement activation could eventually be avoided, alternative strategies are being introduced. These are either based on direct surface functionalization of nanoparticles with complement regulators (such as factor H alone or in combination with factor I), or indirectly via surface functionalization with factor H-binding peptides [2,73,74]. Such functionalization strategies could be expanded to other complement regulators such as soluble domains of CD55 [75]. Another exploratory approach has been cloaking of nanoparticles with plasma membranes of erythrocytes, platelets and macrophages, since plasma membranes of these cells are enriched with a large number of complement regulators such as CD55 and CD59 [76-78]. However, there are many shortcomings with cell membrane cloaking [79,80]. At first instance, effective coating is typically dependent on the quality of cell membrane preparation and preservation of the functionality of the membrane-embedded complement regulators. However, membrane reproducibility is a serious concern due to cell population heterogeneity and phenotype. Regardless, not all nanoparticles might display the acquired cell membrane through its outer bilayer. This population of cell membrane-cloaked nanoparticles could promote proinflammatory reactions [1] and enhance scavenger receptor-mediated immune uptake through exposed phosphatidylserine [81]. Furthermore, phosphatidylserine is also known to trigger complement activation [82].

The emergence of lipid nanoparticle (LNP) mRNA vaccines, which typically contain a small amount of PEG_2000_-lipids, and their multiple administrations are seemingly associated with anti-PEG antibody generation in some individuals [83-86]. Anti-PEG antibodies are further thought to contribute to rare episodes of anaphylactic reactions following LNP vaccination through complement activation [83-85]; however, no convincing evidence, yet, support this hypothesis [87]. Comprehensive studies are needed to purify and characterise these antibodies and map out differences in their properties among different individuals. These attempts could lead to further scrutinisation of their affinity, degree of straining (as in IgM) and clustering (as in IgGs) in relation to PEG characteristics such as the PEG molecular mass, surface density and conformation, thereby offering more insights into the complement activation mechanisms. The low concentration of PEG_2000_-lipids in LNP vaccines strongly suggests the occurrence of surface PEG projections in a “mushroom” conformation [11,12,88], which offers a limited steric protection against protein binding at the interstitial sites and in the lymph. Such non-specific protein binding is expected to interfere with anti-PEG antibody binding. Thus, considering that the majority of these antibodies are expected to exhibit low affinity for PEG [40], we speculate that the most likely mechanism that might drive complement activation is through surface deposition of a few anti-PEG IgG sub-classes that trigger alternative pathway of the complement system [26,30]. This suggestion is consistent with the presence of low concentrations of antibodies and complement pattern recognition molecules at the interstitial sites and in the lymph. It is also not clear as to what extent LNP vaccination raises production of antibodies against lipid components of LNPs and whether these antibodies cross-react with PEG antigen epitopes either directly or through other molecular chaperons. The latter option is analogous to the role of β-2-glycoprotein-1 (apolipoprotein H) as a prerequisite for anti-phospholipid antibody binding to liposomes and complement activation [89]. Nevertheless, a small amount of local complement activation by LNPs could be beneficial and synergistically contribute to the pro-inflammatory properties of ionisable lipids [90-92]. Indeed, the adjuvanticity of the C3d fragment (in fluid phase and surface-bound), which is liberated on complement activation and C3 processing, is well known, particularly in T_H_2 sensitisation, B-cell activation and long-term memory [93-95]. Antibody (IgG) binding to LNPs might also contribute to their clearance by local phagocytic cells through their Fcγ-receptors. Potentially, this could lead either to anaphylaxis or desensitisation, depending on Fcγ receptor type engagement and signalling threshold [96].

Particle engineering initiatives also open avenues for a fundamental complement research. For example, engineered nanoparticles could serve as valuable tools for scrutinising microbial defence mechanisms. Indeed, understanding of the role of particle curvature in an antibody-mediated complement activation has already shed light on some microbial strategies that provide protection against the host immune system [27]. For example, Gram-positive bacteria such as *Staphylococcus aureus* (*S. aureus*) release their peptidoglycan in particulate forms from the cell wall. Analysis of peptidoglycan particles has indicated comparable curvature-related complement activation responses to synthetic nanoparticles through IgM binding, straining and subsequent C1q recruitment [27]. Thus, it appears that the most frequently liberated peptidoglycan particles are in the region of 70–90 nm in size, which serve as decoys by facilitating complement activation through the classical pathway [27]. This provides protection for *S. aureus* through futile conversion of local C3. It should be emphasised that *S. aureus* has a diameter in the order of 1000 nm, thus in an intact cell wall the curvature is unlikely to support complement activation through conformational regulation of the surface-bound IgM of IgG clusters. In parallel, recent understanding that ligand presentation on the 2–20 nm scale may be an important structural feature in regulating C1q and MBL surface binding [53,54] suggests that microbial pattern presentation in the Angstrom-scale arrangement/clusters might be an additional mechanism that confers protection to complement attack [55]. This deserves further exploration.

Finally, advances in nanotechnology are expected to shape our fundamental understanding of inter-related factors that modulate polyvalent interactions among nanoparticles, antibodies and complement proteins on both nanometer and Angstrom scales and point to a wide range of new possibilities for engineering of nanoparticles and surfaces with tuneable complement responses.

## Data Availability

No data was used for the research described in the article.
